# Clavispora mexcalensis sp. nov.: a novel Mexican yeast species isolated from mezcal must with promising polyol and ethanol production from lignocellulose hydrolysates

**DOI:** 10.1099/ijsem.0.007324

**Published:** 2026-11-12

**Authors:** Alejandro Lara-Meléndez, Juan Alfredo Hernández-García, Teresa Ponce-Noyola, Lourdes Villa-Tanaca, Ana C. Ramos-Valdivia, Josefina Barrera-Cortés, Luis M. Salgado

**Affiliations:** 1 Departamento de Biotecnología y Bioingeniería, Centro de Investigación y de Estudios Avanzados del Instituto Politécnico Nacional (Cinvestav). Av. IPN 2508, Zacatenco, Ciudad de México 07360, México; 2 Departamento de Microbiología, Escuela Nacional de Ciencias Biológicas, Instituto Politécnico Nacional, Prolongación de Carpio y Plan de Ayala, Col. Sto. Tomás, 11340 Ciudad de México, México; 3 Centro de Investigación en Ciencia Aplicada y Tecnología Avanzada del Instituto Politécnico Nacional, Colinas del Cimatario, Querétaro 76090, Qro., México

**Keywords:** biorefineries, *Clavispora mexcalensis*, comparative genomics, genome sequencing, mezcal must, non-conventional yeasts

## Abstract

Yeasts are micro-organisms with high biotechnological potential for various industrial processes. Non-conventional yeasts offer advantages over classic *Saccharomyces* yeasts, such as the ability to assimilate pentoses natively. This project aimed to describe and characterize a new yeast species isolated from mezcal must in Oaxaca, México. For its identification, whole-genome sequencing was performed. Phylogenomic analyses (BUSCO, OrthoFinder and IQ-TREE) indicated 99.0% genome completeness of included 2,319 orthologs. Comparative genomic analyses were performed using average nucleotide identity (ANI), calculated with FastANI and core-proteome phylogenomics, complemented by average amino acid identity (AAI) comparisons. Additionally, macro- and micro-morphological analyses (the latter by scanning electron microscopy) were performed. l-arabinose assimilation and thermotolerance were also assessed. The uptake of various sugars, including xylose and arabinose, was evaluated, and the corresponding metabolic pathways were confirmed through experimental and genomic analyses. Phylogenomic analysis, together with FastANI and complementary AAI values, confirmed that, despite its close relationship to *Clavispora lusitaniae* and *Clavispora paralusitaniae*, this strain represents a novel species, for which the name *Clavispora mexcalensis* sp. nov. is proposed. The strain exhibits marked thermotolerance, maintaining substantial growth at 48 °C. It assimilates glucose, d-xylose, l-arabinose and cellobiose as sole carbon sources, and produces xylitol, ethanol and arabitol. Macro- and micro-morphological characteristics are consistent with those of other members of the genus *Clavispora*, although some cells exhibit numerous bud scars. These results expand the known diversity of yeasts associated with mezcal and indicate that *Cl. mexcalensis* sp. nov. is a potential candidate for biotechnological applications requiring high-temperature tolerance or the bioconversion of unconventional sugars.

## Data Availability

The whole-genome shotgun project of *Clavispora mexcalensis* CDBB-L-2031 has been deposited at DDBJ/ENA/GenBank under the BioProject accession number PRJNA1288489 and BioSample accession number SAMN41328489. The individual Internal Transcribed Spacer (ITS) and 28S rRNA gene sequences are available in GenBank under accession numbers PZ593364.1 and PZ671185.1, respectively. The yeast strain described in this study has been deposited in the National Collection of Microbial Strains and Cell Cultures (CDBB), México, under the accession number CDBB-L-2031 and in the Spanish Collection of Type Cultures (CECT) under the accession code CECT 13265ᵀ. All other relevant data are within the manuscript.

## Introduction

Yeasts are the most studied eukaryotic micro-organisms, mainly unicellular fungi, that play a central role in fermentation processes. By converting sugars into alcohol, carbon dioxide and other organic compounds, they are indispensable to produce food, beverages and biofuels. Their ability to metabolize diverse substrates under both aerobic and anaerobic conditions makes them valuable allies in biotechnology [1, 2].

Conventional yeasts such as *Saccharomyces cerevisiae* are model organisms for studying fermentation, but they exhibit limited capacity to assimilate pentose sugars. In contrast, non-conventional yeasts (NCYs), including *Kluyveromyces*, *Candida*, *Pichia* and, more recently, *Clavispora*, offer promising alternatives due to their ability to ferment pentoses and non-glucose sugars. NCYs are increasingly relevant in bioethanol production and xylitol synthesis, and many display enhanced tolerance to heat and stress commonly encountered in industrial fermentations [3]. Beyond energy applications, NCYs are also promising in food biotechnology, given their metabolic diversity, ecological specialization, low biosafety risks and capacity to valorize agro-industrial by-products into food ingredients, additives and bio-based compounds [3–6].

Species of *Clavispora* inhabit diverse ecological niches, including plants, insects and industrial wastes, and are also part of the human microbiota [7, 8]. *Clavispora lusitaniae* is recognized as an opportunistic pathogen, though with relatively low clinical incidence [9]. Members of this genus have also been isolated from fermented products such as mezcal must, pulque, yogurt and tequila [7–10]. These yeasts can metabolize a broad range of sugars (including glucose, galactose, lactose, xylose and cellobiose), making them attractive for biorefinery processes based on saccharified agro-industrial waste and enabling production of ethanol, xylitol and potentially erythritol [11–13].

In this study, the isolation of an unconventional yeast from mezcal must in Oaxaca, México, is reported, within the genus *Clavispora*. Unlike well-characterized *Clavispora* species such as *Cl. lusitaniae* and *Cl. paralusitaniae*, whose genomes range from 12.1 to 12.6 Mb with 4,930–6,100 annotated genes [14–16], our isolate exhibits distinctive genomic features that deviate notably from these species. Whole-genome assembly, annotation and comparative analyses strongly support its classification as a previously uncharacterized – and potentially novel – species. Furthermore, its genetic attributes were evaluated to assess its biotechnological potential, particularly considering its unusual ecological origin.

## Methods

### Strain isolation

The strain was isolated from a 1-ml sample of mezcal must collected from a mezcal distillery (known as palenque) in the town of San Bernardo Mixtepec, municipality of Zimatlán de Álvarez, Oaxaca, México (16° 50′ 00″ N 96° 55′ 00″ W). The sample was plated onto Petri dishes containing Sabouraud Dextrose Agar (SDA) medium. The plates were incubated at 30 °C for 48–72 h until individual colonies appeared. Subsequent isolation of single colonies was performed to ensure purity.

### Yeast growth conditions

*Clavispora mexcalensis* CDBB-L-2031 was routinely cultured on SDA or Yeast Extract Peptone Dextrose (YPD) agar plates and incubated at 30 °C for 48 h. Purity and typical colony morphology were regularly confirmed on solid media.

### Strain deposition

The type strain has been deposited in the National Collection of Microbial Strains and Cell Cultures (CDBB) under the accession code CDBB-L-2031 and the Spanish Collection of Type Cultures (CECT) under the accession code CECT 13265ᵀ.

### Ribosomal DNA analysis and taxonomic re-evaluation

The initial molecular characterization of the isolate was based on the ITS region, which identified it as *Cl. lusitaniae* [17]. To achieve higher taxonomic resolution, the nuclear large subunit rRNA gene (28S nrLSU, D1/D2 domain) was analysed separately (Figs S1 and S2), and a concatenated ITS +28S dataset was generated for multilocus phylogenetic inference. The nt sequences of both ribosomal markers were deposited in GenBank under accession numbers PZ593364.1 (ITS) and PZ671185.1 (28S rRNA gene). Sequence chromatograms were manually inspected and edited using SeaView, and each marker was aligned independently with ClustalX. The ITS and 28S alignments were subsequently concatenated for multilocus analysis. Maximum-likelihood (ML) phylogenetic analyses were performed using IQ-TREE v3.0.1. For each single-locus alignment and each partition of the concatenated dataset, the best-fitting nt substitution model was automatically selected based on the Bayesian Information Criterion (BIC). ML trees were inferred using the selected evolutionary models, and branch support was evaluated with 1,000 SH-like approximate likelihood ratio test (SH-aLRT) replicates and 1,000 nonparametric bootstrap replicates. The concatenated phylogeny included type strains of *Clavispora* and closely related taxa, with appropriate outgroup species.

### Genome sequencing, assembly and annotation

The complete genome of *Cl. mexcalensis* CDBB-L-2031 was sequenced by Novogene (California, USA) using an Illumina NovaSeq PE150 platform, generating paired-end reads (2×150 bp).

Raw reads were quality-checked using FastQC v0.11.9 [18] and trimmed with Trimmomatic v0.39 [19]. The genome was assembled using SPAdes v3.15.5 [20]. To improve contiguity and reduce fragmentation, RagTag v2.1.0 [21] was used for scaffolding and correction against closely related reference genomes. The final assembly comprised 13 scaffolds and 36 contigs, totalling 11.95 Mbp, with a scaffold N50 of 1 Mbp and a gap content of 0.019%. Assembly quality was assessed using QUAST v5.0.2 [22].

Genome completeness was evaluated using BUSCO v6.0.0 [23] in genome mode with the saccharomycetes_odb12 dataset (2,319 orthologs), employing miniprot (v0.18-r281) as the gene predictor. Gene prediction and functional annotation were performed using the Funannotate v1.8.15 pipeline [24], which integrates multiple tools for protein-coding gene prediction and evidence-based refinement. The genome sequence and annotation of *Cl. mexcalensis* sp. nov. have been deposited at DNA Data Bank of Japan (DDBJ)/European Nucleotide Archive (ENA)/GenBank under the accession JBSZHA000000000 (BioProject PRJNA1288489). The native ITS region and the D1/D2 domains of the 28S rRNA gene analysed in section ‘Ribosomal DNA analysis and taxonomic re-evaluation’ were retrieved *in silico* from this assembly, ensuring absolute identity with the corresponding ribosomal regions integrated within the whole-genome shotgun record.

### Phylogenetic and phylogenomic analysis

To evaluate evolutionary relationships and taxonomic boundaries at the whole-genome level, a genomic dataset comprising 67 genome assemblies representing the genus *Clavispora* and related members of the family Metschnikowiaceae, together with our novel isolate CDBB-L-2031, was compiled from the National Center for Biotechnology Information (NCBI) GenBank/RefSeq databases. To minimize topological artefacts and alignment bias associated with highly fragmented assemblies, a stringent quality filter was applied, retaining only genomes containing fewer than 50 scaffolds. For assemblies lacking publicly available structural annotations, coding sequences and predicted proteomes (.*faa* files) were generated *ab initio* using Augustus v3.4.0 with yeast-specific training models. Orthologous gene clusters were identified across the 67 proteomes using OrthoFinder v2.5.5 [25], and the resulting concatenated supermatrix of orthologous proteins (SpeciesTreeAlignment.fa) was used for ML phylogenomic reconstruction in IQ-TREE v3.0.1 [26] (Linux x86_64, 32-thread parallelization). The best-fitting aa substitution model (Q.yeast +I+R10) was selected automatically by ModelFinder according to the BIC. Branch support was assessed using 1,000SH-aLRT replicates and 1,000 ultrafast bootstrap replicates.

For quantitative proteome-wide taxonomic delimitation, average amino acid identity (AAI) was calculated for all pairwise comparisons among the 67 proteomes using the mutual best-hit algorithm implemented in EzAAI v1.2.4 [27]. Reciprocal best hits were identified with DIAMOND [28], generating a comprehensive pairwise AAI matrix. The resulting similarity values were organized into a 67×67 matrix. To visualize genomic relationships and assess species boundaries relative to the accepted operational threshold for yeasts (~95–96% AAI), a hierarchically clustered heatmap (clustermap) with an unweighted pair group method with arithmetic mean (UPGMA) dendrogram was generated using Python visualization libraries. Hierarchical clustering and annotated heatmaps were produced with the function in Seaborn v0.12.2, together with Matplotlib v3.7.1; the complete AAI dataset and clustered heatmap are provided as Supplementary Materials 3 and 4. Average nucleotide identity (ANI) was calculated using FastANI [29] with the genome of strain CDBB-L-2031 and 29 publicly available genomes representing *Clavispora* and closely related taxa. The focal comparison included 21 genomes assigned to *Cl. lusitaniae,* including the type strain CBS 6936ᵀ (GCA_001673695.2), and the type strain of *Cl. paralusitaniae*, NYNU 161120ᵀ (GCA_022058765.1). Pairwise comparisons were performed in both query–reference orientations using a *k*-mer size of 16 and a fragment length of 3,000 nts. The aligned fraction (AF) was calculated as the number of mapped query fragments divided by the total number of query fragments. Both directional ANI and AF values were retained. Hierarchical clustering of the ANI heatmap was performed using UPGMA based on the dissimilarity measure *d*=100−ANI; this dendrogram was not interpreted as an evolutionary phylogeny.

### Carbon source assimilation assay

The assimilation of different carbon sources was assessed in 24-well microplate-based growth assays. The inoculum was prepared from an overnight culture grown in YPD medium. Cells were harvested by centrifugation, washed twice with sterile saline solution (0.85%) and resuspended in sterile deionized water to the initial volume. The starting inoculum corresponded to ~0.7 OD_₆₀₀_ at time zero (T0). Each well contained 2 ml of yeast nitrogen base (YNB) medium supplemented with one of 18 different carbon sources at a final concentration of 1% (wt/v). To prevent cell sedimentation during incubation, a sterile glass bead (4 mm diameter) was added to each well. Control conditions included YNB without any carbon source (negative control) and YNB supplemented with 1% (wt/v) glucose (positive control). Each condition was assayed in triplicate.

Cultures were incubated at 30 °C at 150 r.p.m. for 72 h. Growth was monitored by measuring OD at 600 nm (OD_₆₀₀_) at 0, 24, 48 and 72 h using a CLARIOstar microplate reader (BMG Labtech, Germany). For samples that exceeded the linear detection range of the instrument, a 1 : 20 dilution was performed before re-measurement.

### Ethanol tolerance assay

Ethanol tolerance was evaluated using a sterile 24-well microplate-based growth assay in YNB medium. The inoculum was prepared as reported above. Each well was prepared with YNB medium (1× final concentration), the appropriate volume of absolute ethanol, and finally the inoculum to adjust to 2 ml. Two independent experimental series were conducted: (i) YNB supplemented with ethanol only and (ii) YNB supplemented with ethanol and 1% (wt/v) glucose. Ethanol concentrations were prepared to yield nominal (theoretical) values of 1, 3, 5, 7, 9, 11, 13, 15 and 17% (v/v). The actual concentrations obtained, based on volumetric calculations, were 0.99, 2.99, 5.01, 6.98, 8.99, 11.01, 13.02, 14.99 and 17.00% (v/v), respectively, plus a control condition without ethanol. All conditions were assayed in triplicate.

Plates were incubated at 30 °C and 150 r.p.m. for 72 h. Growth was monitored by measuring OD_₆₀₀_ at 0, 24, 48 and 72 h using a microplate reader (CLARIOstar, BMG Labtech, Germany).

### Scanning electron microscopy sample preparation

For scanning electron microscopy (SEM) analysis, *Cl. mexcalensis* cells were harvested by centrifugation. The cells were then washed twice with PBS pH 7.2 and fixed for 1 h with 2.5% glutaraldehyde in PBS. Fixed cells were subsequently adhered to poly-l-lysine-covered coverslips. Coverslips with the *Cl. mexcalensis* samples were washed in the same buffer and then dehydrated through a graded series of ethanol concentrations (50–100%). For drying, samples were incubated three times for 10 min each with hexamethyldisilazane (HMDS; 692 Polysciences, Warrington, PA). After the excess HMDS was eliminated, samples were left to air-dry for 30 min. Finally, the dried samples were gold-sputtered using a Denton Vacuum Desk V (Denton Vacuum, Moorestown, NY, USA) and examined under a Scanning Electron Microscope JSM-6510LV (JEOL Ltd., Tokyo, Japan).

## Results

### ITS and D1/D2-based molecular analyses

Phylogenetic analysis based on concatenated sequences of the ITS region (ITS1–5.8S–ITS2) and the D1/D2 domains of the 28S rRNA gene placed strain CDBB-L-2031 within the family Metschnikowiaceae, clustering within the *Clavispora* clade (Fig. 1). However, the high sequence conservation of these standard ribosomal markers resulted in poor phylogenetic resolution within the *Cl. lusitaniae* species complex, with strain CDBB-L-2031 clustering closely with related taxa and forming short internal branches. Consequently, species-level relationships could not be resolved confidently using either single-locus or concatenated ribosomal datasets. This lack of resolution highlights the limited discriminatory power of conventional ribosomal barcodes in complex systems and underscores the need for genome-scale analyses to establish robust taxonomic boundaries.

**Fig. 1.**
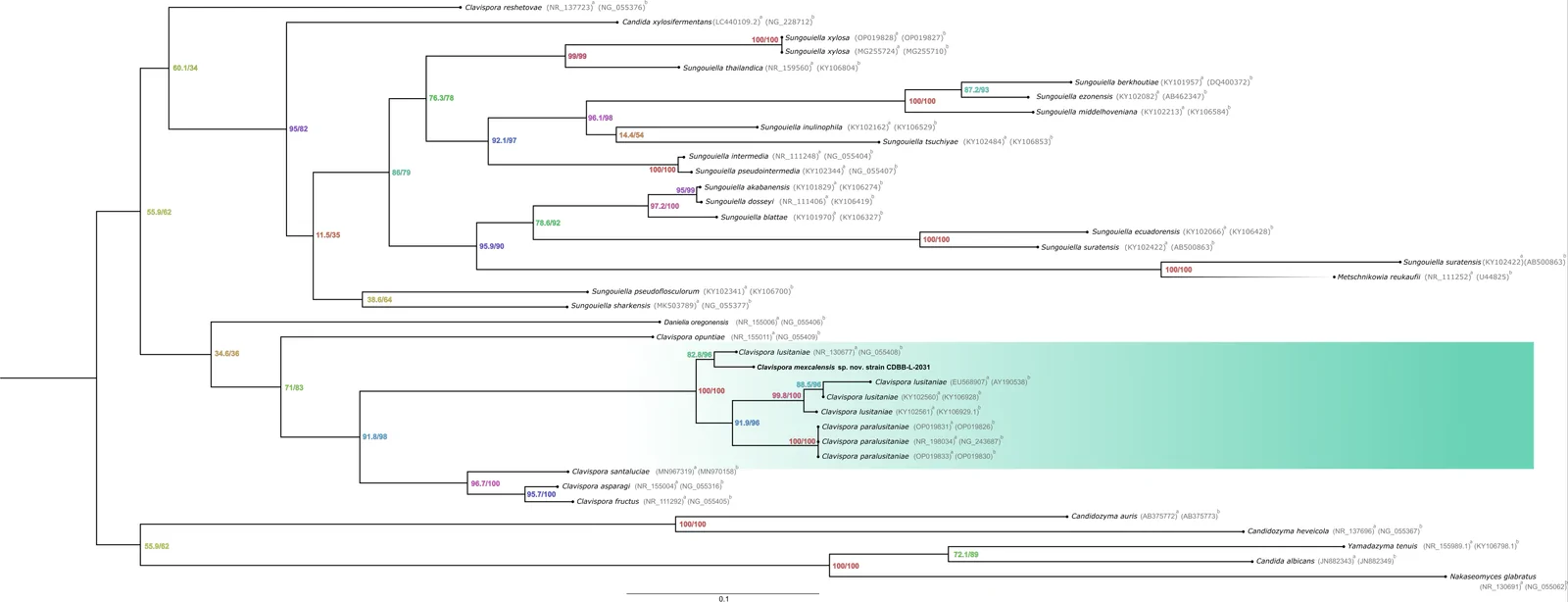
ML phylogenetic tree based on concatenated sequences of the ITS region (ITS1–5.8S–ITS2) and the D1/D2 domains of the LSU 28S rRNA gene, showing the position of *Cl. mexcalensis* CDBB-L-2031 relative to its closest taxa. Bootstrap support values ≥50% are indicated at the nodes. The tree was rooted using an outgroup comprising *Candidozyma auris*, *Candidozyma heveicola*, *Yamadazyma tenuis*, *Candida albicans* and *Nakaseomyces glabrata*. Branch tip labels include the species name followed by the NCBI accession numbers for the ITS and D1/D2 regions in parentheses. Superscript letters a and b denote the ITS and D1/D2 sequences, respectively.

### Phylogenomic relationships

Phylogenomic analysis of the core proteome from the expanded 67-genome dataset robustly resolved the placement of *Cl. mexcalensis* strain CDBB-L-2031 within the *Clavispora sensu stricto* clade (Fig. 2). The final genome assembly of *Cl. mexcalensis* showed high contiguity and completeness, with a total size of 11.95 Mbp distributed across 13 scaffolds and a scaffold N50 of 1 Mbp. Genome completeness was estimated at 99.0 % (C:99.0 %) using BUSCO v6.0.0 with the saccharomycetes_odb12 dataset, identifying 2,294 (98.9%) orthologs as complete and single-copy, with only 1.0% (23 orthologs) missing.

**Fig. 2.**
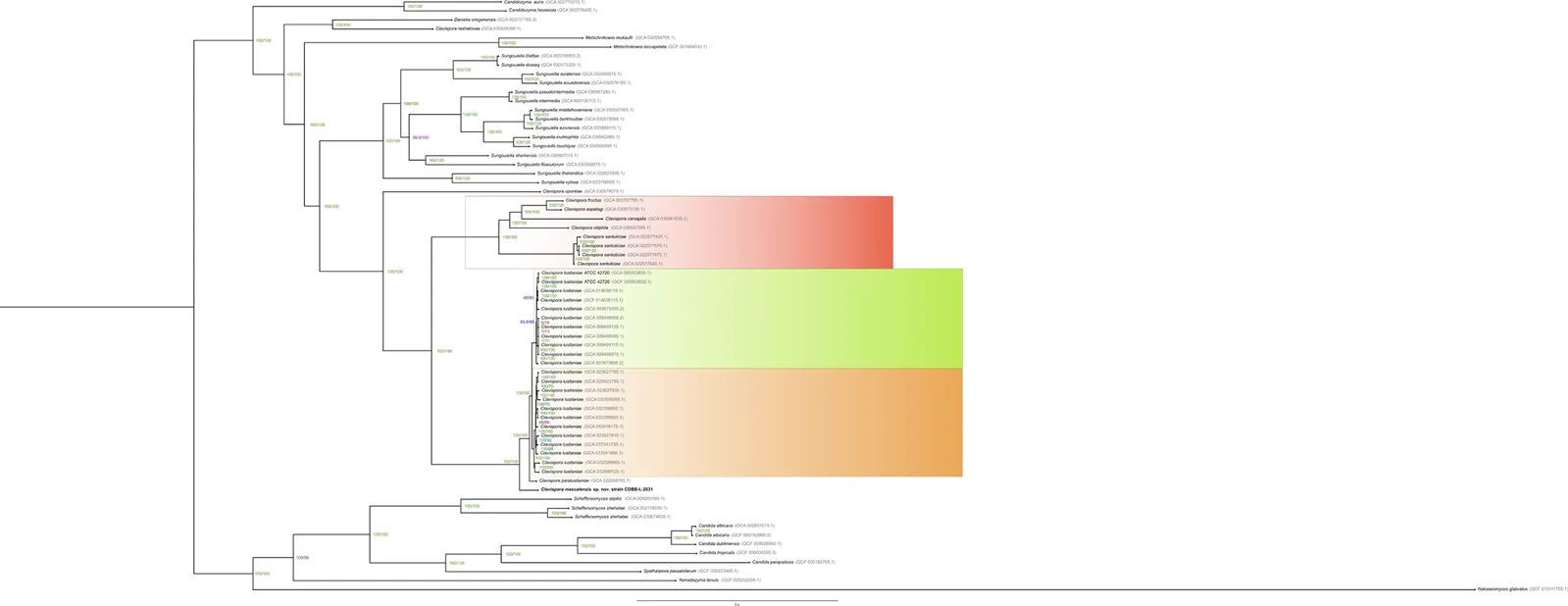
ML phylogenomic reconstruction of *Cl. mexcalensis* CDBB-L-2031 and related taxa based on core-proteome analysis. The dataset includes available whole-genome sequences from the *Cl. lusitaniae* species complex, closely related *Clavispora* species and representative Metschnikowiaceae outgroups. Numbers at the nodes indicate ultrafast bootstrap support values based on 1,000 replicates.

The novel species formed a distinct sister lineage to the core clade comprising *Cl. lusitaniae* and *Cl. paralusitaniae*, with maximal branch support (100% SH-aLRT and 100% ultrafast bootstrap) (Fig. 2). This well-supported proteome-based phylogeny provides strong evidence that strain CDBB-L-2031 represents an evolutionarily independent lineage, clearly distinct from both the core species and the more deeply diverged lineages within the genus.

### Genome-based similarity metrics

The bidirectional comparison between CDBB-L-2031 and the *Cl. lusitaniae* type strain CBS 6936ᵀ yielded ANI values of 88.6038 and 88.6216%, with AFs of 93.5127 and 92.9003%, respectively (symmetric mean: 88.6127%). Across all 21 evaluated *Cl. lusitaniae* genomes, the symmetric ANI values with CDBB-L-2031 ranged from 88.2922 to 88.6478% (median: 88.6062%). By contrast, the 210 pairwise ANI values within *Cl. lusitaniae* ranged from 96.7732 to 99.9997% (median: 98.4693%). The type strain of *Cl. paralusitaniae* showed ANI values of 94.9224–95.3774% against the 21 *Cl*. *lusitaniae* genomes, whereas its ANI with CDBB-L-2031 was 88.5390%. Therefore, CDBB-L-2031 is genomically distinct from all evaluated *Cl. lusitaniae* genomes and *Cl. paralusitaniae*, showing a marked genomic discontinuity (>8.1% points) relative to the lowest intra-species ANI value observed among *Cl. lusitaniae* (Table 1, Fig. 3). Proteome-wide AAI analysis showed a fully concordant pattern and was retained as complementary evidence (Figs S3 and S4).

**Table 1.** Bidirectional FastANI results for strain CDBB-L-2031 against 21 genomes assigned to *Cl. lusitaniae*

Strain (genome accession)	ANI M→L (%)	AF M→L (%)	ANI L→M (%)	Mean ANI (%)
ATCC 42720 (GCA_000003835.1)	88.6120	94.2419	88.6004	88.6062
CBS 6936^T^ (GCA_001673695.2)	88.6038	93.5127	88.6216	88.6127
L17 (GCA_003675555.2)	88.3468	91.6269	88.2377	88.2922
P1 (GCA_009498055.1)	88.5691	94.6191	88.5493	88.5592
P2 (GCA_009498075.1)	88.6072	93.9653	88.5809	88.5941
P3 (GCA_009498095.1)	88.5811	94.2419	88.5470	88.5641
P5 (GCA_009498115.1)	88.5993	93.9904	88.5468	88.5730
P4 (GCA_009498135.1)	88.5774	94.3173	88.5472	88.5623
FDAARGOS_655 (GCA_014636115.1)	88.6357	94.3676	88.6243	88.6300
P1110A (GCA_023627755.1)	88.6564	93.5881	88.6239	88.6402
P8014A (GCA_023627815.1)	88.6320	94.2419	88.6134	88.6227
P4013B (GCA_023627835.1)	88.6349	93.8396	88.6058	88.6204
P1110B (GCA_025503755.1)	88.6545	93.8899	88.6117	88.6331
DH3710_C_M08-1 (GCA_032358895.1)	88.6383	81.1919	88.5785	88.6084
DH3706_C_L04 (GCA_032358955.1)	88.6304	74.7548	88.6258	88.6281
UCDFST_82–606.2 (GCA_032599065.1)	88.5700	83.6309	88.5400	88.5550
UCDFST_76–31 (GCA_032599085.1)	88.6226	83.1531	88.6242	88.6234
UCDFST_80–11 (GCA_032599125.1)	88.5830	77.0179	88.5480	88.5655
R121-14C (GCA_037041735.1)	88.6622	94.2167	88.6333	88.6478
R6405 (GCA_037041895.1)	88.6347	93.4373	88.5742	88.6044
KM1 (GCA_050916175.1)	88.5904	94.3928	88.5659	88.5781

M denotes CDBB-L-2031, and L denotes the corresponding *Cl. lusitaniae* genome. Reciprocal AF values, aligned-fragment counts and the complete all-versus-all ANI matrix are provided in the Supplementary Data. The mean was used only to obtain a symmetric matrix for visualization; the directional values remain the primary FastANI outputs.

**Fig. 3.**
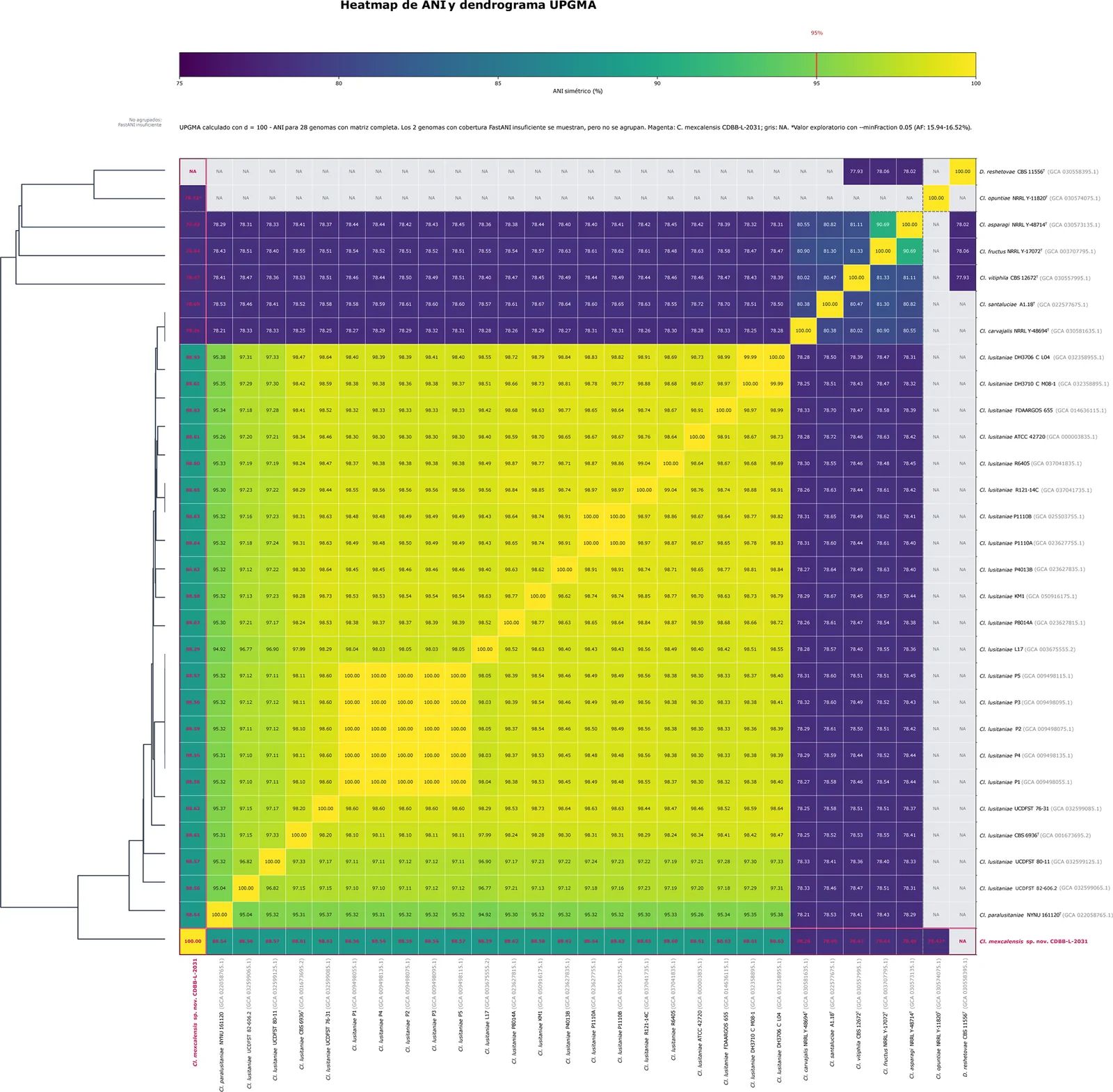
Heatmap of symmetric FastANI values and descriptive UPGMA clustering among *Clavispora* and related genomes. *Cl. mexcalensis* CDBB-L-2031 is highlighted in magenta; type strains are indicated with a superscript T. Grey cells denote na comparisons.

Furthermore, core-proteome phylogenomic analysis (Fig. 2), corroborated by proteome-wide AAI comparisons (Fig. S3), revealed two notable patterns within the genus.

#### Cryptic lineage

Phylogenomic reconstruction resolved a distinct, well-supported monophyletic lineage within the *Cl. lusitaniae* species complex (Fig. 2). In concordance with this topology, members of this lineage shared >98.4% internal AAI but only 95.0–96.2% AAI with *Clavispora lusitaniae sensu stricto* (type strain ATCC 42720) (Fig. S3). Together, these phylogenomic and proteomic data support the recognition of this candidate cryptic species, here provisionally designated *Clavispora cryptolusitaniae*.

#### Genus-level polyphyly

At a broader taxonomic scale, the core-proteome phylogeny demonstrated that species currently assigned to *Clavispora* are distributed across deeply divergent lineages (Fig. 2). Whereas *Cl. mexcalensis*, *Cl. paralusitaniae* and the candidate cryptic lineage formed a strongly supported monophyletic core together with the type species *Cl. lusitaniae*, a separate lineage comprising *Clavisporaopuntiae*, *Clavispora santaluciae*, *Clavispora carvajalis*, *Clavispora reshetovae* and *Clavispora vitiphila* branched independently of the *Cl*. *sensu stricto* clade (Fig. 2). Consistent with this phylogenetic separation, pairwise AAI values between this lineage and the *Cl*. *sensu stricto* core ranged from 74.0 to 76.5% (Fig. S3).

### Taxonomy

*Clavispora mexcalensis* Lara-Meléndez, Hernández-García and Ponce-Noyola, sp. nov. (Fig. 4).

**Fig. 4.**
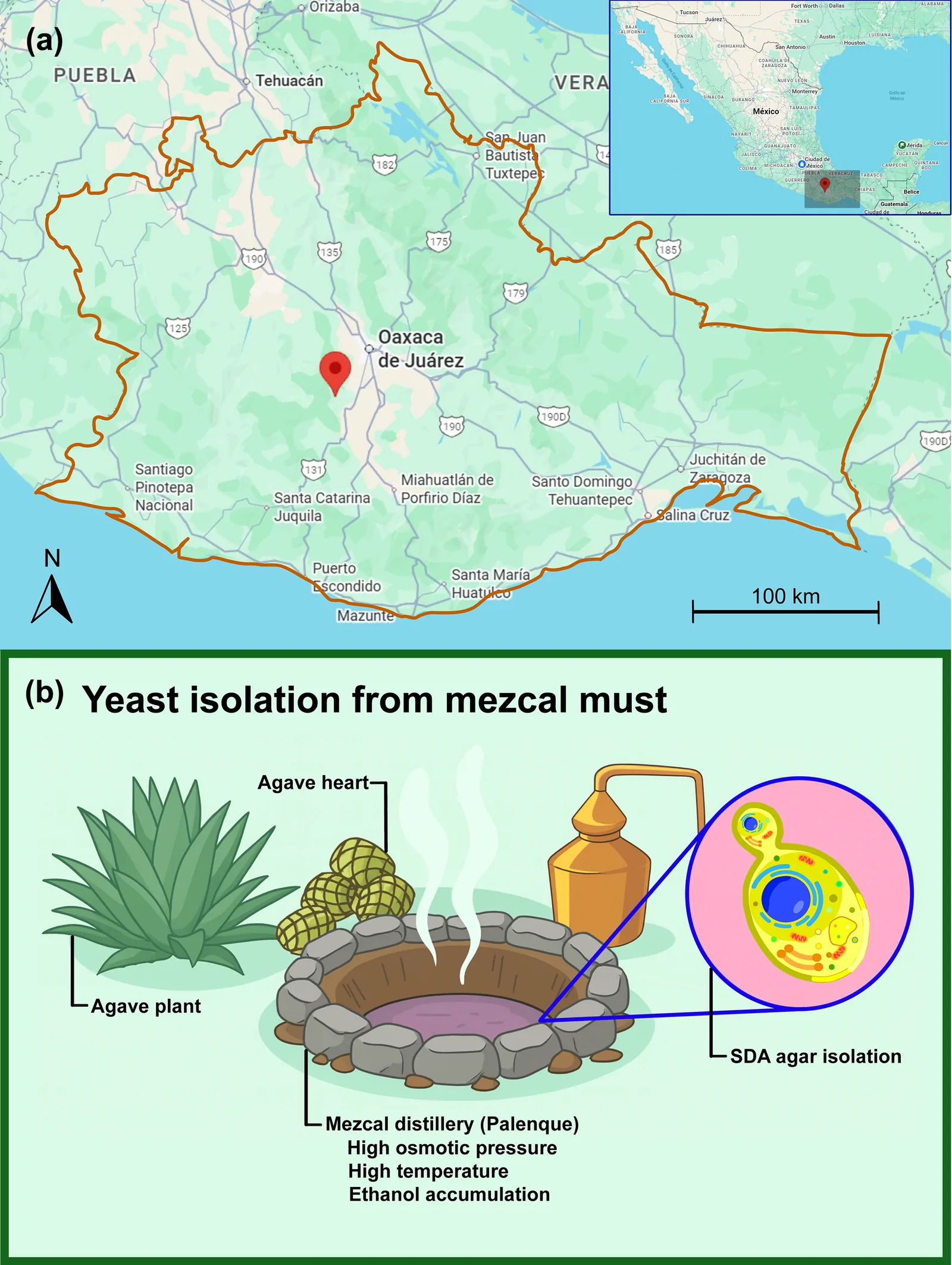
Geographical origin and ecological niche of the type strain *Cl. mexcalensis* CDBB-L-2031. (**a**) Main map displaying the State of Oaxaca with a red pin marking the specific isolation locality in San Bernardo Mixtepec (16° 50′ N, 96° 55′ W); the upper inset shows a map of México, where a dark grey rectangle indicates the location of the enlarged area. (**b**) Schematic representation of the isolation process from *Agave* fermentation must (Mezcal), illustrating the environmental conditions (high osmolarity and ethanol accumulation) associated with the strain’s stress tolerance.

MycoBank number: MB 861415

Etymology: *mex.ca.len’sis*. N.L. fem. adj. *mexcalensis*, about the Mexican mezcal-producing region; an arbitrary geographical epithet derived from México, the country of origin of the type strain and mezcal*,* the traditional fermented agave beverage from which the type strain was isolated, combined with the Latin suffix *-*ensis, denoting origin or association with a place.

Diagnosis: Differs from *Clavispora lusitaniae* and *Clavispora paralusitaniae* by its ability to assimilate l-arabinose and by its capacity for robust growth at 48 °C.

Type: México: Oaxaca, municipality of Zimatlán de Álvarez, San Bernardo Mixtepec (16.8333° N, 96.9167° W; altitude 1,746 m). Isolated from mezcal distillery must in a traditional palenque, June 2002, by Leonor Patricia Rodríguez Pascual.

Holotype CDBB-L-2031ᵀ, preserved in a metabolically inactive state at the National Collection of Microbial Strains and Cell Cultures (Cinvestav, México); ex-type strain CECT 13265ᵀ.

GenBank/ENA/DDBJ: JBSZHA000000000 (genome sequence), PZ593364.1 (ITS region sequence) and PZ671185.1 (28S rRNA gene sequence).

Description: Asexual reproduction by multilateral budding; cells are elliptical to broadly ovoid, 3–4×1.5–2.5 µm, occurring singly or in sporadic clusters of up to four cells. Budding cells exhibit up to six polar bud scars, appearing as raised annular rings or shallow depressions (0.75–1.25 µm in diameter). Older cells may display subtle surface wrinkling. No pseudohyphae, true hyphae, ascospores or prominent extracellular matrix components were observed on YPD or SDA media. Colonies on YPD agar after 48 h at 30 °C are circular, creamy white, convex, smooth with a butyrous and entire margin, reaching 1–2 mm in diameter. Cultures emit a distinct sweet and ethanolic odour, resembling that of a fermented beverage. No pseudohyphae, true hyphae or ascospores were observed under the conditions assayed (Fig. 5).

**Fig. 5.**
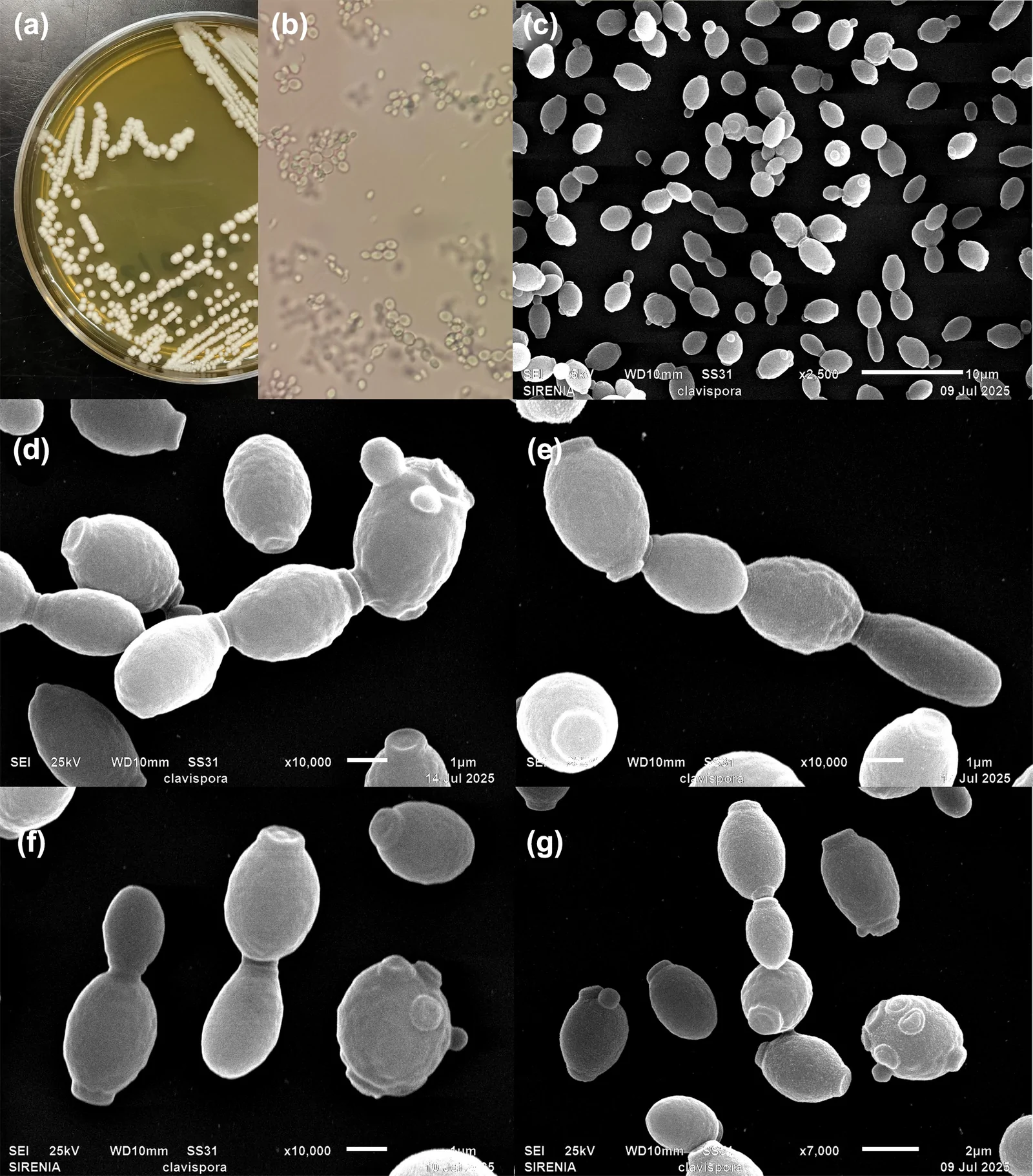
Polyphasic morphological characterization of *Cl. mexcalensis* sp. nov. CDBB-L-2031. (**a**) Colonial morphology on YPD agar after 48 h incubation at 30 °C, showing circular, creamy white and convex colonies with a smooth surface. (**b**) Light microscopy of vegetative cells showing elliptical shapes. (**c**) SEM overview of the yeast population displaying uniform elliptical to broadly ovoid cells. (d, e) SEM micrographs illustrating cell chains that remain physically attached after budding; Panel d specifically details a cell with four distinct bud scars (white arrowheads) and a nascent bud (white arrow). (f, g) High-magnification images of older cells, distinguished by an increased number of bud scars and prominent surface wrinkling compared to younger cells. Scale bars: 10 µm (**c**); 1 µm (d, f); 2 µm (**g**).

The genome sequence and annotation of *Cl. mexcalensis* sp. nov. have been deposited at DDBJ/ENA/GenBank under the accession JBSZHA000000000 (BioProject PRJNA1288489) and BioSample SAMN41328489.

Physiology: Growth occurs at 30–48 °C, with optimal growth at 30 °C. Fermentation of glucose is positive.

Assimilation of carbon sources: Positive for glucose, d-xylose, l-arabinose, sucrose, glycerol, ethanol, mannitol, sorbitol, cellobiose, l-rhamnose, mannose, fructose and acetate. Weak or slow assimilation for d-arabinose, d-galactose, raffinose and trehalose. Negative for lactose and myo-inositol. Ethanol tolerance up to 9% (v/v) (Tables 2 and 3).

**Table 2.** Growth of *Cl. mexcalensis* sp. nov. on different carbon sources, and its comparison with related species

	*Cl. mexcalensis*	*Cl. lusitaniae* [12, 13, 30]	*Cl. paralusitaniae* [16]
Glucose	+	+	+
d-Xylose	+	+	+
l-Arabinose	+	V	−
d-Arabinose	+ (S)	−	−
Lactose	−	+	−
d-Galactose	+ (S)	+	+
Sucrose	+	V	+
Glycerol	+	+	+
Ethanol	+	+	−
Manitol	+	+	+
Sorbitol	+	+	nd
Cellobiose	+	+	+
myo-inositol	−	−	−
Raffinose	+ (S)	+	−
l-Rhamnose	+	V	+
Trehalose	+ (S)	+	+
Mannose	+	nd	nd
Fructose	+	nd	nd
Acetate	+	nd	nd

+ (positive), − (negative), S (slow growth seen until 48 h), V (variable consumption) and nd (no data available).

**Table 3.** Growth of *Cl. mexcalensis* sp. nov. under increasing ethanol concentrations (0–17%) in YNB medium, with and without 1% (wt/v) glucose

	Ethanol	Ethanol+glucose
0%	−	+
1%	+	+
3%	+	+
5%	+	+
7%	+ (S)	+ (S)
9%	+ (S)	+ (S)
11%	−	−
13%	−	−
15%	−	−
17%	−	−

+ (positive), – (Negative), S (slow; growth detected up to 48 h)

Genomic accession numbers: The whole-genome sequence and annotation of *Cl. mexcalensis* sp. nov. have been deposited in DDBJ/ENA/GenBank under accession JBSZHA000000000 (BioProject PRJNA1288489; BioSample SAMN41328489). The corresponding ITS region and D1/D2 domains of the 28S rRNA gene were also deposited separately in GenBank under accession numbers PZ593364.1 and PZ671185.1, respectively.

### Physiological characterization

#### Carbon source assimilation profile

The complete assimilation profile is detailed in Table 2. Notably, *Cl. mexcalensis* demonstrated metabolic versatility by assimilating pentoses (d-xylose and l-arabinose) and disaccharides (cellobiose and sucrose). While assimilation of glucose and d-xylose was rapid, growth on l-arabinose, d-arabinose and d-galactose was slower, becoming evident only after 48 h. This profile distinguishes the new species from *Cl. paralusitaniae*, which is unable to assimilate l-arabinose or raffinose. Unlike *Cl. lusitaniae*, *Cl. mexcalensis* assimilated d-arabinose but failed to grow on lactose.

### Ethanol tolerance and thermotolerance

The yeast strain exhibited tolerance to ethanol concentrations up to 9% (v/v) in YNB medium (Table 3). Although growth was detectable at 5% ethanol after 24 h, adaptation to higher concentrations (7 and 9%) required 48 h of incubation. No growth was observed at concentrations ≥11%. Glucose supplementation (1% wt/v) enhanced biomass production but did not extend the maximum tolerance limit beyond 9%.

*Cl. mexcalensis* exhibited significant growth over a wide temperature range (30–48 °C). Optimal growth occurred at 30 °C, reaching a maximum OD_₆₀₀_ of 31.8 after 48 h. Remarkably, the strain maintained detectable growth even at 48 °C, a thermal resilience trait not typically reported for closely related *Clavispora* species (data not shown).

### Metabolic pathway inference

In the annotated genome, we identified genes involved in the final steps of the synthesis of important biotechnological compounds. These enzymes include alcohol dehydrogenase 1, erythrose reductase, xylose reductase and d-arabitol-2-dehydrogenase.

The physiological capacity of *Cl. mexcalensis* to utilize various carbon sources, particularly pentoses like d-xylose and l-arabinose, was experimentally confirmed (Table 2). This experimental evidence, along with the genomic annotation, was used to reconstruct a metabolic pathway map focused on biotechnologically relevant metabolites: xylitol, ethanol, d-erythritol and d-arabitol (Fig. 6). The map illustrates the pentose assimilation pathways, specifically xylose and l-arabinose utilization, showing how both sugars feed into the pentose phosphate pathway (PPP), which then interfaces with glycolysis and ultimately leads to ethanolic fermentation.

**Fig. 6.**
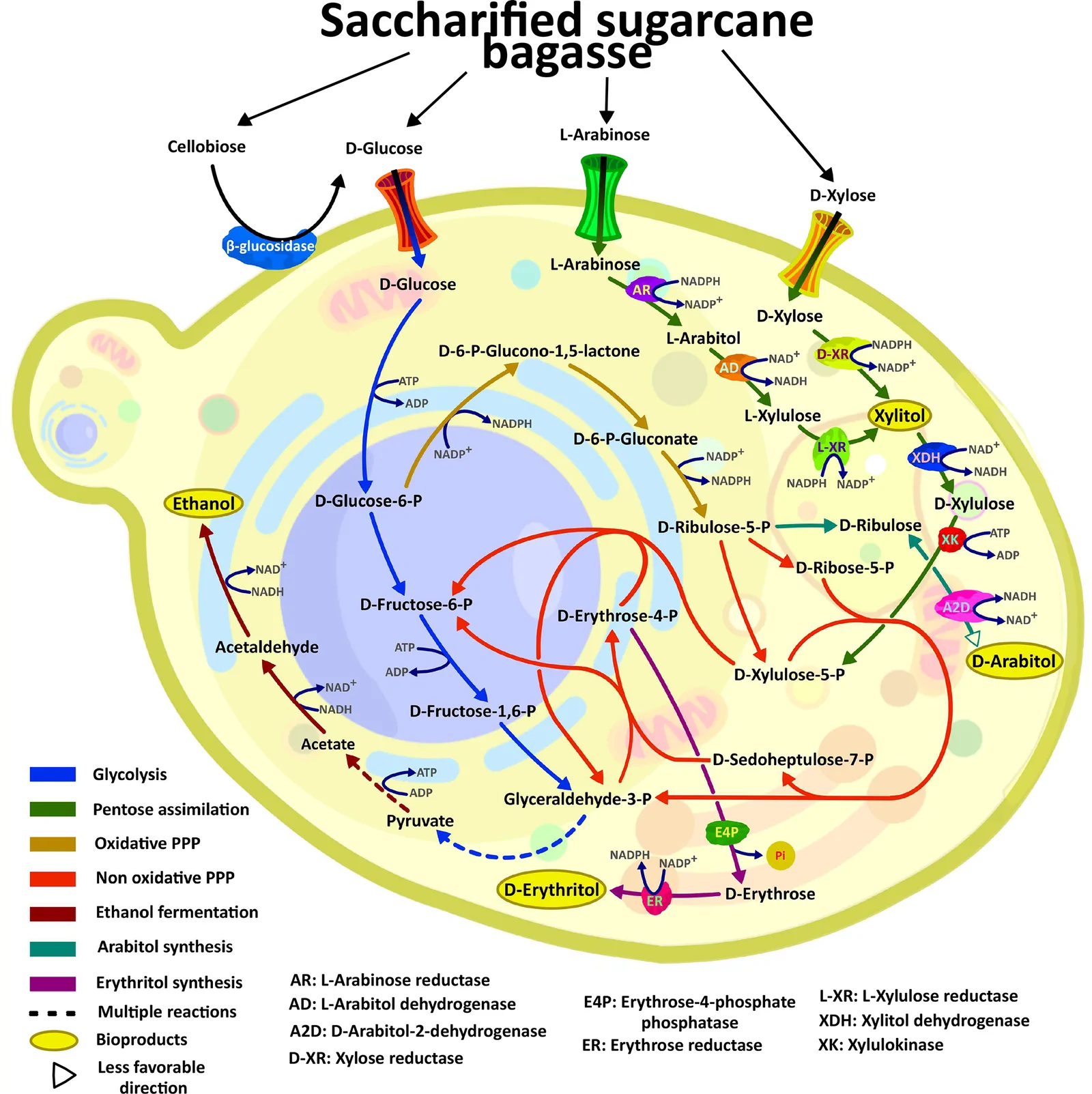
Metabolic pathways for biotechnologically valuable products in *Cl. mexcalensis* CBDD-L-2031, inferred from genome functional annotation and supported by experimental evidence using saccharified sugarcane bagasse.

## Discussion

Species delimitation within the *Clavispora* genus has historically presented challenges due to phenotypic variability. The genus was erected by Rodrigues de Miranda [30] following the discovery of a strain of complementary mating type in a citrus peel sample. However, identification based solely on growth characteristics has proven unreliable. For instance, the isolate UWOPS92-308.1 from crushed agave, confirmed as a member of the genus [31], failed to assimilate sucrose and other key sugars, differing from the type description.

In accordance with the identification difficulties mentioned above, the initial characterization of strain CDBB-L-2031 using conventional molecular markers (ITS1, ITS2, 5.8S, 18S and 28S rDNA gene) identified it as *Cl. lusitaniae* [5]. Subsequent phylogenetic analysis based on ribosomal sequences positioned the strain within the *Cl. lusitaniae/paralusitaniae* clade (Fig. 1). However, reference sequences of *Cl. lusitaniae* (e.g. GenBank accessions NR_130677 and NG_055408) clustered with our isolate, but the relevant nodes received variable bootstrap support, indicating that these ribosomal markers alone do not provide sufficient resolution for confident species-level discrimination. High sequence conservation of ribosomal DNA loci within the *Cl. lusitaniae* complex has been widely documented [10, 12, 31]. Although the ITS region and the 28S rRNA gene (D1/D2 domains) are widely used for yeast identification, their limited sequence divergence among closely related taxa frequently results in short branch lengths and overlapping phylogenetic clusters, thereby reducing their discriminatory power for species-level identification [12, 31].

To overcome these limitations and further assess the taxonomic placement of strain CDBB-L-2031, a comprehensive phylogenomic analysis incorporating 67 genome assemblies was performed. Whole-proteome-based phylogenomic reconstruction fully resolved the relationships within the *Cl. lusitaniae* complex (Fig. 2), placing strain CDBB-L-2031 as a distinct sister lineage to the *Cl. lusitaniae–Cl. paralusitaniae* clade with maximal statistical support (100% SH-aLRT; 100% ultrafast bootstrap), and genome-based similarity metrics corroborated this placement.

Expanded ribosomal sampling reflects the close phylogenetic affinity of CDBB-L-2031 to the *Cl. lusitaniae* complex (Fig. 1). However, the genome-wide analysis demonstrates that CDBB-L-2031 falls outside the observed ANI range of *Cl. lusitaniae*: its maximum symmetric ANI with any evaluated *Cl. lusitaniae* genome was 88.6478%, whereas the minimum ANI observed among *Cl. lusitaniae* genomes was 96.7732% (Table 1, Fig. 3). This marked discontinuity (>8.1% points) is substantially greater than that observed between *Cl. lusitaniae* and the accepted species *Cl. paralusitaniae* (94.92–95.38% ANI) [32]. Furthermore, the high AFs (>92.9%) observed in the direct bidirectional comparison with the type strain CBS 6936ᵀ argue against an artefact resulting from small or unrepresentative conserved fractions of the assemblies. Complementary proteome-wide AAI comparisons (91.48–92.53% with *Cl. lusitaniae* and 92.97% with *Cl. paralusitaniae*) showed complete concordance with this genomic divergence (Fig. S3), further supporting the conclusion that strain CDBB-L-2031 represents a distinct species rather than intraspecific variation.

Beyond supporting the recognition of *Cl. mexcalensis*, the expanded 67-genome phylogenomic framework provided additional insights into genomic relationships within the genus. Hierarchical clustering of the AAI matrix identified a distinct, highly cohesive cluster within the *Cl. lusitaniae* complex, sharing >98.4% internal AAI but only 95.0–96.2% AAI relative to *Cl. lusitaniae sensu stricto* (type strain ATCC 42720). This pattern is consistent with the presence of a cryptic genomic lineage within the complex. To facilitate future taxonomic studies of this group, we propose the provisional designation *Cl. cryptolusitaniae* for this candidate cryptic species.

At the genus level, our phylogenomic analyses indicate that *Clavispora*, as currently circumscribed, comprises deeply divergent lineages, as previously suggested by multigene phylogenies [33, 34]. Whereas *Cl. mexcalensis*, *Cl. paralusitaniae*, *Cl. cryptolusitaniae* (candidate cryptic species) and *Cl. lusitaniae* form a well-supported clade centred on the type species, *Cl. reshetovae, Cl. vitiphila, Cl. opuntiae*, *Cl. santaluciae* and *Cl. carvajalis* constitute a separate, deeply divergent lineage. Pairwise AAI values between this lineage and the core *Clavispora* clade range from 74.0 to 76.5%, overlapping the values commonly associated with intergeneric divergence in yeasts (~75–80% AAI). Although formal taxonomic revision is beyond the scope of the present study, these lineages may represent distinct genera. Pending comprehensive taxonomic revision, we provisionally refer to them as the ‘Neoclavispora’ and ‘Opuntienella’ clades.

The carbon source assimilation profile of *Cl. mexcalensis* (Table 2) revealed a broad metabolic versatility that is distinct from *Cl. paralusitaniae* and, overall, like *Cl. lusitaniae*. This capacity to use substrates such as glucose, cellobiose, xylose and l-arabinose is consistent with its origin in mezcal must, an environment rich in lignocellulose-derived compounds. Unlike *Cl. paralusitaniae*, the new species assimilated both l-arabinose and raffinose, though growth on raffinose was barely detectable, indicating limited hydrolysis of the trisaccharide. Growth on d-arabinose, galactose and trehalose became evident only after 48 h, at levels lower than xylose but higher than raffinose. Compared to *Cl. lusitaniae*, *Cl. mexcalensis* shared most assimilation traits but differed in l-arabinose, l-rhamnose and lactose and uniquely assimilated d-arabinose. This versatility, supported by the presence of a complete metabolic network for pentose utilization, highlights its potential for bioconversion and biorefinery applications.

*Cl. mexcalensis* also tolerated ethanol concentrations up to 9% (v/v), a level consistent with natural mezcal fermentations, where ethanol rarely exceeds 10–12% [35, 36]. This tolerance likely confers a competitive advantage in such environments. Comparisons show that *Cl. lusitaniae* strains display variable tolerance, from ceasing growth above ~9% to reaching 12–15% depending on strain and context [37–39]. The tolerance observed in *Cl. mexcalensis* thus falls within this range, consistent with ethanol resistance as a common trait in the clade. Relative to *S. cerevisiae*, which averages ~14% (≈112 g l^−1^) but varies markedly among strains [40], *Cl. mexcalensis* exhibits lower tolerance, comparable to several non-*Saccharomyces* yeasts in complex fermentations. This trait, together with its genomic divergence and physiological features, supports its recognition as a novel species and underscores its ecological relevance and potential in agave-based fermentations.

Macroscopic morphology of *Cl. mexcalensis* colonies resembled that of *Cl. paralusitaniae* (white to cream, buttery, convex with entire margins; [16]) and was comparable to descriptions of *Cl. lusitaniae* colonies [41, 42], indicating a generally conserved appearance among *Clavispora* species. These robust macroscopic observations contribute to the polyphasic characterization of the new species.

Thermotolerance tests showed robust growth of *Cl. mexcalensis* up to 48 °C, with an optimum at 30 °C. This surpasses many industrial yeasts such as *S. cerevisiae*, which typically grows optimally at 32–34 °C and ceases at ~45 °C [43, 44], even under optimized conditions [45]. Within *Clavispora*, variation exists: some *Cl. lusitaniae* strains grow at 50 °C [46], while others do not grow above 37–40 °C [47, 48]. *Cl. paralusitaniae* grows at 37 °C but not at 40 °C [16], and *Cl. santaluciae* does not grow above 35 °C [12]. The ability of *Cl. mexcalensis* to grow strongly at 48 °C positions it among the more thermotolerant members of the clade and above many conventional industrial strains.

SEM revealed *Cl. mexcalensis* cells to be elliptical to broadly ovoid (3–4×1.5–2.5 µm) (Fig. 5B–G). Cells showed up to six bud scars, mostly at the poles, indicating a consistent budding pattern. Subtle surface wrinkling was observed, particularly in scarred cells, possibly reflecting variations in turgor or minor irregularities. No pseudohyphae, true hyphae, spores or extracellular matrix were observed (Fig. 5D–G). These features align with descriptions of other *Clavispora* species. *Cl. paralusitaniae* has ovoid to elliptical cells (2–7×2–8 µm), occurring singly or in pairs with multilateral budding [16]. *Cl. lusitaniae* cells range from 1.5 to 6×2.5–10 µm, can form pseudohyphae, and invade agar [30, 49]. The dimensions of *Cl. mexcalensis* fall within the lower end of these ranges, suggesting a smaller and more homogeneous morphology, which may represent a distinctive feature under the conditions tested. The microscopic characterization confirmed asexual reproduction through multilateral budding, with no pseudohyphae or ascospores observed under the tested conditions. These traits support its placement within *Clavispora* and provide phenotypic evidence for its novelty, although additional sporulation tests are still needed. Future studies could explore the sexual life cycle and mating-type characteristics of *Cl. mexcalensis*, aspects that were outside the scope of the present taxonomic and metabolic characterization.

Genome annotation and assimilation tests also revealed metabolic potential consistent with production of ethanol, erythritol, xylitol and d-arabitol. Genes encoding key enzymes – alcohol dehydrogenase 1 (ADH1), erythrose reductase, xylose reductase (XYL1) and d-arabitol-2-dehydrogenase (ARD1) – were identified. Their presence suggests complete pathways, consistent with experimental evidence of ethanol production from glucose and cellobiose and xylitol from xylose [17, 50]. These findings reinforce the potential of *Cl. mexcalensis* for bioconversion applications and biorefinery processes and serve as a basis for further validation by gene expression, enzyme activity and metabolite profiling studies.

The genomic and physiological features of *Cl. mexcalensis* sp. nov. are consistent with the metabolic traits previously reported for strain CDBB-L-2031, which in earlier studies had been identified as *Cl. lusitaniae*. These prior works – conducted before the present taxonomic reassessment – already revealed their remarkable capability to metabolize multiple lignocellulose-derived sugars and to produce industrially relevant metabolites.

Ochoa-Chacón *et al*. [5] demonstrated that *Cl. lusitaniae* CDBB-L-2031, now identified as *Cl. mexcalensis,* produced ethanol from glucose and cellobiose and xylitol from xylose under microaerobic conditions, reaching yields of ethanol of 0.45 g g⁻¹ and 0.34 g g⁻¹ from glucose and cellobiose, respectively, and a xylitol yield of 0.42 g g⁻¹ from xylose. Moreover, the *XYL1* and *XYL2* genes were up-regulated by xylose and down-regulated by glucose. This supports the genomic basis for polyol biosynthesis, indicating that these genes play a crucial role in the process. The up-regulation and down-regulation of these genes in response to different sugars is a key finding, as it suggests a regulatory mechanism that could be exploited for industrial applications.

In a more recent study, Guzmán-Hernández *et al.* [50] evaluated the same strain (now recognized as *Cl. mexcalensis*) in saccharified sugarcane bagasse under different aeration conditions. They demonstrated co-production of ethanol and xylitol through sequential anaerobic/microaerobic phases, achieving 31.8 g l⁻¹ ethanol with a yield of 0.49 g g⁻¹ under anaerobiosis. In contrast, xylitol titre was 14.3 g l⁻¹ under microaerobic conditions. These results were associated with maximum xylose reductase (XR) and xylitol dehydrogenase (XDH) activities (2.9 and 1.7 U mg⁻¹, respectively) and strong induction of *the XYL1* and *XYL2* genes (>30 fold for *XYL1*).

Taken together, these studies substantiate the biotechnological potential of *Cl. mexcalensis* sp. nov., whose genome encodes complete pathways for alcohol and polyol metabolism (e.g. *ADH1*, *XYL1* and *ARD1*). By integrating prior experimental evidence with the genomic framework presented here, it reinforces the view that *Cl. mexcalensis* represents a promising NCY for lignocellulosic biorefineries and the sustainable production of ethanol, xylitol and erythritol.

## Conclusion

This study describes *Cl. mexcalensis* sp. nov., a novel yeast species isolated from mezcal must in Oaxaca, México, and establishes its taxonomic status using a polyphasic framework. Although standard ribosomal markers provided limited phylogenetic resolution, genome-scale analyses – including core-proteome phylogenomics and FastANI pairwise comparisons (ANI<89%, with a marked >8.1 percentage-point discontinuity relative to *Cl. lusitaniae*) – clearly distinguish this species from its closest relatives, *Cl. lusitaniae* and *Cl. paralusitaniae*. These findings highlight the decisive role of genome-wide approaches in delineating species boundaries within closely related yeast complexes. More broadly, the distinctiveness of *Cl. mexcalensis* suggests that diversity within the *Cl. lusitaniae* clade is currently underestimated and that isolates assigned to this complex may include additional undescribed cryptic species.

Beyond its taxonomic significance, *Cl. mexcalensis* sp. nov. exhibits a phenotypic and metabolic profile indicative of substantial biotechnological potential. The strain shows marked thermotolerance, sustaining growth at temperatures up to 48 °C, exceeding those of many established industrial yeasts. It also displays metabolic flexibility, including the assimilation of diverse carbon sources such as the pentoses d-xylose and l-arabinose, which are relevant for lignocellulosic biomass valorization. Genomic and experimental analyses further support the presence of biosynthetic pathways for the production of value-added compounds, including ethanol, xylitol and arabitol. Together, these features expand the diversity of yeasts associated with traditional Mexican fermentations and position *Cl. mexcalensis* as a promising candidate for biorefineries and other industrial applications that require thermotolerance and efficient use of non-conventional substrates.

### Declaration of generative AI and AI-assisted technologies in the manuscript preparation process

During the preparation of this work, the authors used ChatGPT and Gemini to assist in drafting and rephrasing sentences for better scientific communication. Additionally, Microsoft Copilot (DALL-E 3) was used to generate specific graphic elements (illustrations of agave plants and distillation equipment) incorporated into Fig. 4. The final composition and scientific labelling of the figure were performed by the authors. After using this tool/service, the authors reviewed and edited the content as needed and take full responsibility for the content of the published article.

## Supplementary material

10.1099/ijsem.0.007324 Supplementary Material 1.

## Abbreviations

AAI: average amino acid identity
ADH1: alcohol dehydrogenase 1
AF: aligned fraction
ANI: average nucleotide identity
ARD: d-arabitol-2-dehydrogenase
BIC: Bayesian Information Criterion
HMDS: hexamethyldisilazane
ML: maximum-likelihood
NCY: non-conventional yeast
OD_₆₀₀_: OD at 600 nm
PPP: pentose phosphate pathway
SDA: Sabouraud Dextrose Agar
SEM: scanning electron microscopy
SH-aLRT: SH-like approximate likelihood ratio test
UPGMA: unweighted pair group method with arithmetic mean
XYL: xylose reductase
YNB: yeast nitrogen base
YPD: Yeast Extract Peptone Dextrose

## References

[1] Albuquerque TL de, da Silva IJ Jr, de Macedo GR, Rocha MVP (2014). Biotechnological production of xylitol from lignocellulosic wastes: A review. Process Biochemistry.

[2] Ndubuisi IA, Amadi CO, Nwagu TN, Murata Y, Ogbonna JC (2023). Non-conventional yeast strains: Unexploited resources for effective commercialization of second generation bioethanol. Biotechnol Adv.

[3] Binati RL, Salvetti E, Bzducha-Wróbel A, Bašinskienė L, Čižeikienė D (2021). Non-conventional yeasts for food and additives production in a circular economy perspective. FEMS Yeast Res.

[4] Wendland J (2019). Special issue: non-conventional yeasts: genomics and biotechnology. Microorganisms.

[5] Ochoa-Chacón A, Ramos-Valdivia AC, Poggi-Varaldo HM, Villa-Tanaca L, Martinez A (2022). Fermentation performance of a Mexican native *Clavispora lusitaniae* strain for xylitol and ethanol production from xylose, glucose and cellobiose. Enzyme Microb Technol.

[6] Lara-Meléndez A, Guzmán-Hernández D, Montiel-Cruz J, Ponce-Noyola T (2023). Yeast Biotechnol Biofuels Sustain.

[7] Cooper CR (2011). The Yeasts.

[8] Hommel RK (2014). Encycl. Food Microbiol.

[9] Gabaldón T, Naranjo-Ortíz MA, Marcet-Houben M (2016). Evolutionary genomics of yeast pathogens in the Saccharomycotina. FEMS Yeast Res.

[10] Pérez-Brito D, Magaña-Alvarez A, Lappe-Oliveras P, Cortes-Velazquez A, Torres-Calzada C (2015). Genetic diversity of *Clavispora lusitaniae* isolated from Agave fourcroydes Lem, as revealed by DNA fingerprinting. J Microbiol.

[11] Shukla R, Hemansi G, Kumar G, Shukla S, Saini JK (2023). Biorefinery potential of newly isolated yeast *Clavispora lusitaniae* for co-production of erythritol and ethanol. Biomass Conv Bioref.

[12] Drumonde-Neves J, Čadež N, Reyes-Domínguez Y, Gallmetzer A, Schuller D (2020). *Clavispora santaluciae* f.a., sp. nov., a novel ascomycetous yeast species isolated from grapes. Int J Syst Evol Microbiol.

[13] Fan G, Liu P, Chang X, Yin H, Cheng L (2021). Isolation and Identification of a High-Yield Ethyl Caproate-Producing Yeast From *Daqu* and Optimization of Its Fermentation. Front Microbiol.

[14] NCBI (2025). *Clavispora lusitaniae* ATCC 42720 genome assembly asm383v1. https://www.ncbi.nlm.nih.gov/datasets/genome/GCF_000003835.1/.

[15] NCBI (2025). *Clavispora lusitaniae* genome assembly asm949805v1. https://www.ncbi.nlm.nih.gov/datasets/genome/GCA_009498055.1/.

[16] Chai C-Y, Li Y, Yan Z-L, Hui F-L (2022). Phylogenetic and genomic analyses of two new species of *Clavispora* (Metschnikowiaceae, Saccharomycetales) from Central China. Front Microbiol.

[17] Ochoa-Chacón A, Martinez A, Poggi-Varaldo HM, Villa-Tanaca L, Ramos-Valdivia AC (2022). Xylose metabolism in bioethanol production: *Saccharomyces cerevisiae* vs non-*Saccharomyces* Yeasts. Bioenerg Res.

[18] Schmieder R, Edwards R (2011). Quality control and preprocessing of metagenomic datasets. Bioinformatics.

[19] Bolger AM, Lohse M, Usadel B (2014). Trimmomatic: a flexible trimmer for Illumina sequence data. Bioinformatics.

[20] Bankevich A, Nurk S, Antipov D, Gurevich AA, Dvorkin M (2012). SPAdes: a new genome assembly algorithm and its applications to single-cell sequencing. J Comput Biol.

[21] Alonge M, Lebeigle L, Kirsche M, Jenike K, Ou S (2022). Automated assembly scaffolding using RagTag elevates a new tomato system for high-throughput genome editing. Genome Biol.

[22] Gurevich A, Saveliev V, Vyahhi N, Tesler G (2013). QUAST: quality assessment tool for genome assemblies. Bioinformatics.

[23] Manni M, Berkeley MR, Seppey M, Simão FA, Zdobnov EM (2021). BUSCO Update: novel and streamlined workflows along with broader and deeper phylogenetic coverage for scoring of eukaryotic, prokaryotic, and viral genomes. Mol Biol Evol.

[24] Palmer JM, Stajich J (2020). Funannotate v1.8.1: Eukaryotic genome annotation, Zenodo.

[25] Emms DM, Kelly S (2019). OrthoFinder: phylogenetic orthology inference for comparative genomics. Genome Biol.

[26] Minh BQ, Schmidt HA, Chernomor O, Schrempf D, Woodhams MD (2020). Corrigendum to: IQ-TREE 2: new models and efficient methods for phylogenetic inference in the genomic era. Mol Biol Evol.

[27] Kim D, Park S, Chun J (2021). Introducing EzAAI: a pipeline for high throughput calculations of prokaryotic average amino acid identity. J Microbiol.

[28] Buchfink B, Reuter K, Drost H-G (2021). Sensitive protein alignments at tree-of-life scale using DIAMOND. Nat Methods.

[29] Jain C, Rodriguez-R LM, Phillippy AM, Konstantinidis KT, Aluru S (2018). High throughput ANI analysis of 90K prokaryotic genomes reveals clear species boundaries. Nat Commun.

[30] Rodrigues de Miranda L (1979). *Clavispora*, a new yeast genus of the Saccharomycetales. Antonie van Leeuwenhoek.

[31] Lachance MA, Daniel HM, Meyer W, Prasad GS, Gautam SP (2003). The D1/D2 domain of the large-subunit rDNA of the yeast species *Clavispora lusitaniae* is unusually polymorphic. FEMS Yeast Res.

[32] Lachance MA, Lee DK, Hsiang T (2020). Delineating yeast species with genome average nucleotide identity: a calibration of ANI with haplontic, heterothallic *Metschnikowia* species. Antonie van Leeuwenhoek.

[33] Tsui CKM, Daniel HM, Robert V, Meyer W (2008). Re-examining the phylogeny of clinically relevant *Candida* species and allied genera based on multigene analyses. FEMS Yeast Res.

[34] Kurtzman CP, Mateo RQ, Kolecka A, Theelen B, Robert V (2015). Advances in yeast systematics and phylogeny and their use as predictors of biotechnologically important metabolic pathways. FEMS Yeast Res.

[35] Pérez-Hernández E, González-Hernández JC, Chávez-Parga Ma del C (2022). Optimization of *Agave cupreata* juice fermentation process for mezcal production using statistical experimental design. Food Sci Technol.

[36] Ávila-Reyes SV, Jiménez-Aparicio AR, Melgar-Lalanne G, Fajardo-Espinoza FS, Hernández-Sánchez H (2025). Mezcal: a review of chemistry, processing, and potential health benefits. Foods.

[37] Martín-Rendón E, Jiménez J, Benítez T (1989). Ethanol inhibition of *Saccharomyces* and *Candida* enzymes. *Curr Genet*.

[38] Duan Z-F, Han M-Y, Niu J-L, Zhao J-R, Li W-W (2024). Evolution of fermented grain yeast communities in strong-flavored baijiu and functional validation of yeasts that produce superior-flavored substances. J Sci Food Agric.

[39] Zhao Y, Liu J, Wang H, Gou F, He Y (2024). Advancements in fermented beverage safety: isolation and application of *Clavispora lusitaniae* Cl-p for ethyl carbamate degradation and enhanced flavor profile. Microorganisms.

[40] Arroyo-López FN, Salvadó Z, Tronchoni J, Guillamón JM, Barrio E (2010). Susceptibility and resistance to ethanol in *Saccharomyces* strains isolated from wild and fermentative environments. Yeast.

[41] Garnoeva R, Dinkova V (2025). Fungal empyema thoracis associated with *Clavispora lusitaniae*—First report in a domestic cat. *JoF*.

[42] Miller NS, Dick JD, Merz WG (2006). Phenotypic switching in *Candida lusitaniae* on copper sulfate indicator agar: association with amphotericin B resistance and filamentation. J Clin Microbiol.

[43] Salvadó Z, Arroyo-López FN, Guillamón JM, Salazar G, Querol A (2011). Temperature adaptation markedly determines evolution within the genus *Saccharomyces*. Appl Environ Microbiol.

[44] García-Ríos E, López-Malo M, Guillamón JM (2014). Global phenotypic and genomic comparison of two *Saccharomyces cerevisiae* wine strains reveals a novel role of the sulfur assimilation pathway in adaptation at low temperature fermentations. BMC Genomics.

[45] Almagro A, Prista C, Castro S, Quintas C, Madeira-Lopes A (2000). Effects of salts on *Debaryomyces hansenii* and *Saccharomyces cerevisiae* under stress conditions. Int J Food Microbiol.

[46] Fan G, Liu P, Chang X, Yin H, Cheng L (2021). Isolation and Identification of a High-Yield Ethyl Caproate-Producing Yeast From *Daqu* and Optimization of Its Fermentation. Front Microbiol.

[47] Limtong S, Kaewwichian R (2015). The diversity of culturable yeasts in the phylloplane of rice in Thailand. *Ann Microbiol*.

[48] Casagrande Pierantoni D, Giuliano S, Conti A, Corte L, Angelini J (2024). Phenotypical differences at the physiological and clinical level between two genetically closely related *Clavispora lusitaniae* strains isolated from patients. *JoF*.

[49] Zhang J, Silao FGS, Bigol UG, Bungay AAC, Nicolas MG (2012). Calcineurin is required for pseudohyphal growth, virulence, and drug resistance in *Candida lusitaniae*. PLoS One.

[50] Guzmán-Hernández D, Ramos-Valdivia AC, Poggi-Varaldo HM, Barrera-Cortés J, Cristiani-Urbina E (2025). Ethanol and Xylitol Co-Production by *Clavispora lusitaniae* growing on saccharified sugar cane bagasse in anaerobic/microaerobic conditions. *Fermentation*.

