# Supplementary material for: Clavispora mexcalensis sp. nov.: a novel Mexican yeast species isolated from mezcal must with promising polyol and ethanol production from lignocellulose hydrolysates
Source: Int J Syst Evol Microbiol. 2026 Nov 12;76(11):007324. doi: 10.1099/ijsem.0.007324 (PMC13759130; doi:10.1099/ijsem.0.007324)
Supplement: Supplementary file 1 [file ijsem-76-07324-s001.pdf]

Supplementary Material for: Revised Manuscript IJSEM-S-25-00882R2 *Clavispora mexcalensis* sp. nov: A novel Mexican yeast species isolated from mezcal must with promising polyol and ethanol production from lignocellulose hydrolysates.

Alejandro Lara-Meléndez[1], Juan Alfredo Hernández-García[2], Teresa Ponce-Noyola[1*], Lourdes Villa-Tanaca[2], Ana C. Ramos-Valdivia[1], Josefina Barrera-Cortés[1], Luis M. Salgado[3].

Caption:

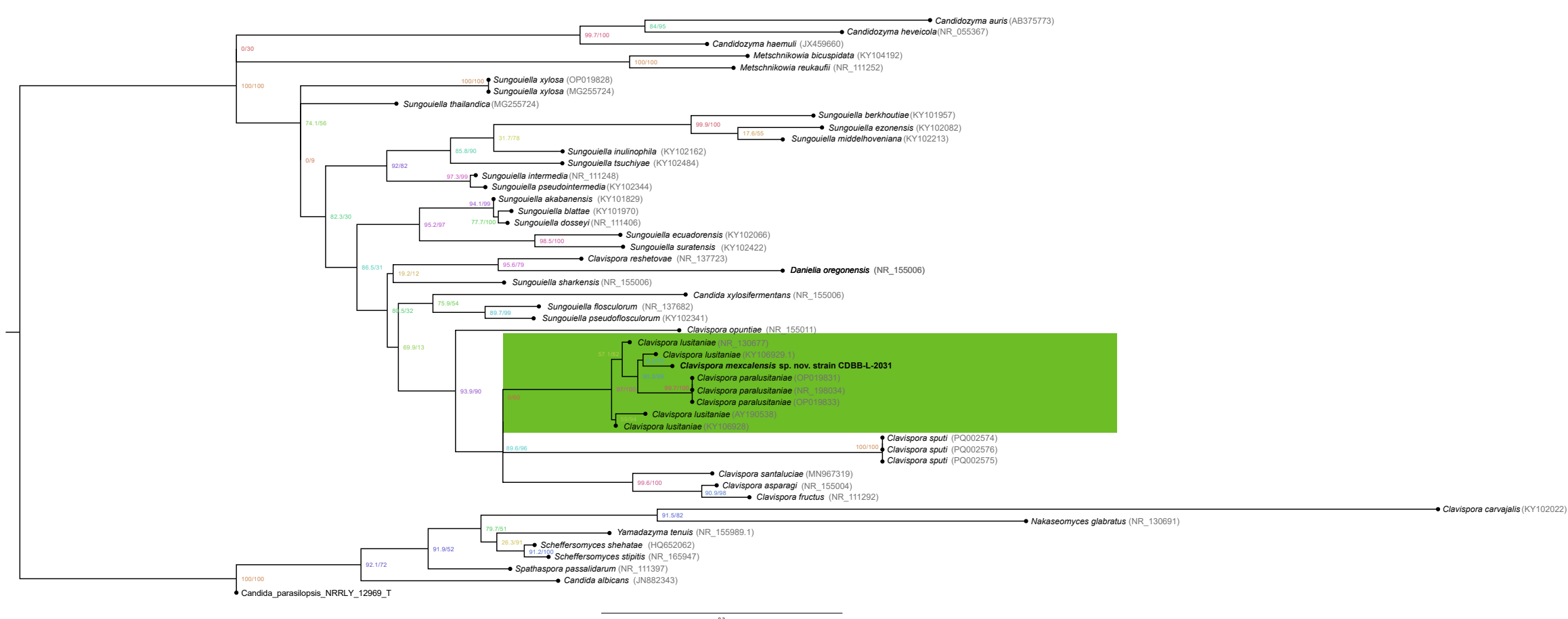

Fig. S1. Phylogenetic tree of D1/D2 sequences. Maximum-likelihood phylogenetic tree based on the D1/D2 domains of the large subunit (26S) rRNA gene, illustrating the evolutionary relationships of *Clavispora mexcalensis* CDBB-L-2031. The tree was inferred using IQ-TREE v2.1.2 with automatic model selection (ModelFinder Plus) and 1,000 ultrafast bootstrap replicates. Support values (SH-aLRT/Ultrafast Bootstrap) are

indicated at the nodes. Rooting and outgroup selection are identical to those described in Fig. S2. Scale bar represents substitutions per nucleotide position.

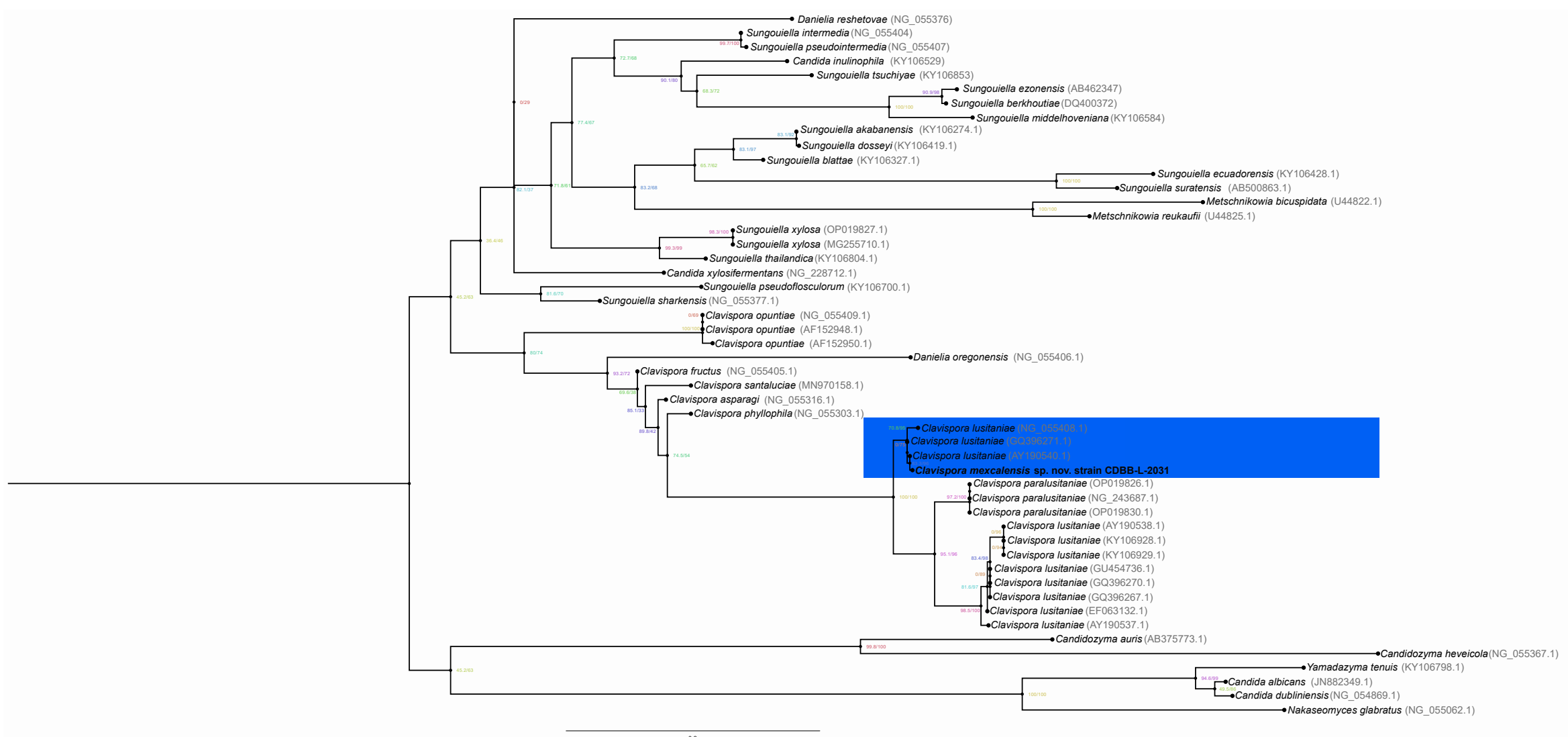

Fig. S2. Phylogenetic tree of ITS sequences. Maximum-likelihood phylogenetic tree based on the internal transcribed spacer (ITS) region sequences, showing the position of *Clavispora mexcalensis* CDBB-L-2031 relative to other members of the *Metschnikowiaceae* family. The tree was constructed using IQ-TREE v2.1.2 with 1,000 ultrafast bootstrap replicates. Numbers at nodes represent SH-aLRT/Ultrafast Bootstrap support values. The analysis was rooted with a clade comprising different Candida-relative genera members.

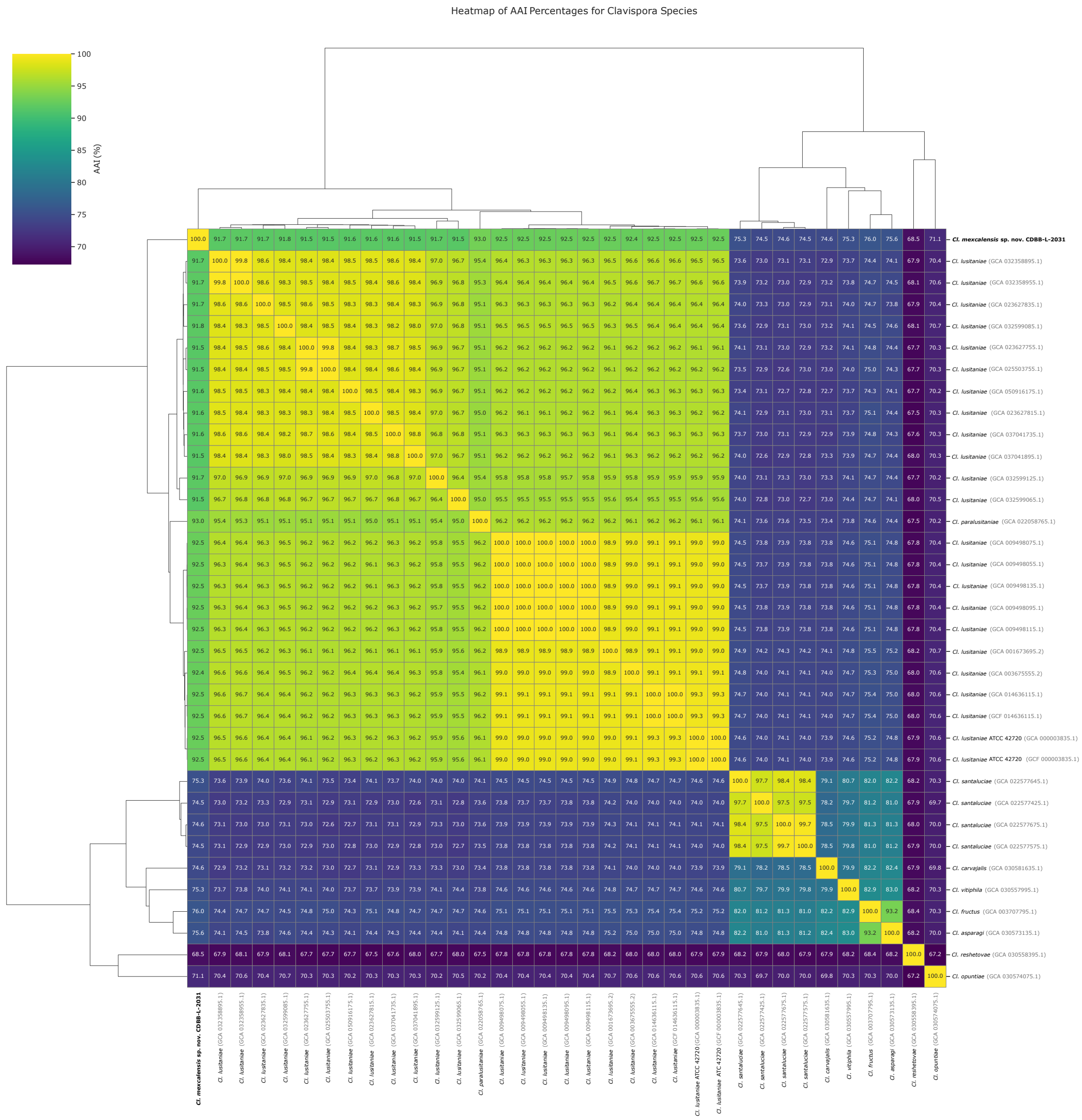

Fig. S3. Average amino acid identity (AAI) heatmap comparing *Clavispora mexcalensis* CDBB-L-2031 with closely related species of the genus *Clavispora*. Pairwise AAI values were calculated using EzAAI v1.2.4. Higher AAI values (typically ≥95%) are generally consistent with conspecific genomes, whereas lower values support species-level genomic divergence.

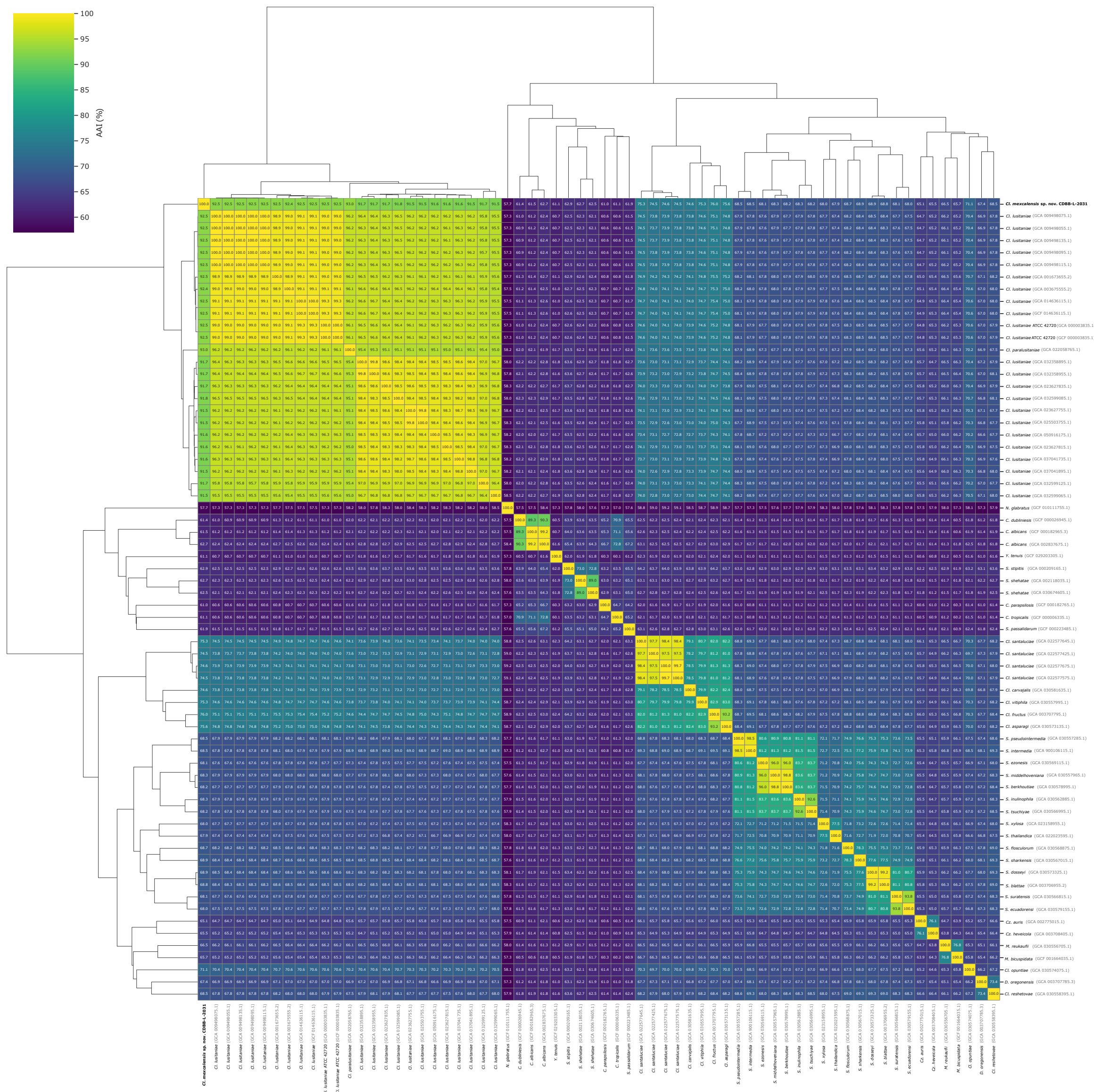

Fig. S4. Extended average amino acid identity (AAI, %) heatmap with hierarchical clustering dendrograms illustrating genomic relationships between *Clavispora mexcalensis* CDBB-L-2031 and a broad representation of ascomycetous yeast species. Hierarchical clustering on both axes reflects pairwise AAI values computed across

complete proteomes using EzAAI v1.2.4. The color gradient scale (60–100%) represents the percentage of amino acid identity.

BUSCO data:

BUSCO version is: 6.0.0

The lineage dataset is: saccharomycetes_odb12 (Creation date: 2025-07-01, number of genomes: 75, number of BUSCOs: 2319)

Summarized benchmarking in BUSCO notation for file /home/schifosi/Documentos/alex_cinves/04_ragtag_cleaned_masked/Clavispora_final_masked.fasta

BUSCO was run in mode: euk_genome_min

Gene predictor used: miniprot

***** Results: *****

C:99.0%[S:98.9%,D:0.0%],F:0.0%,M:1.0%,n:2319,E:13.9%

2295 Complete BUSCOs (C) (318 contain internal stop codons)

2294 Complete and single-copy BUSCOs (S)

1 Complete and duplicated BUSCOs (D)

1 Fragmented BUSCOs (F)

23 Missing BUSCOs (M)

2319 Total BUSCO groups searched

Assembly Statistics:

13	Number of scaffolds
36	Number of contigs
11949596	Total length
0.019%	Percent gaps
1 Mbp	Scaffold N50
834 kbp	Contigs N50

Dependencies and versions:

hmmsearch: 3.4

bbtools: None

miniprot_index: 0.18-r281

miniprot_align: 0.18-r281
